# Resolving morphogenesis into quantifiable cell behaviours

**DOI:** 10.1242/dev.199794

**Published:** 2022-11-14

**Authors:** Jeremy B. A. Green

**Affiliations:** Centre for Craniofacial Regeneration and Biology, King's College London, Guy's Campus, London SE1 9RT, UK

**Keywords:** Cell behaviours, Growth, Morphogenesis, Quantitative biology

## Abstract

Morphogenesis is extremely diverse, but its systematic quantification to determine the physical mechanisms that produce different phenotypes is possible by quantifying the underlying cell behaviours. These are limited and definable: they consist of cell proliferation, orientation of cell division, cell rearrangement, directional matrix production, cell addition/subtraction and cell size/shape change. Although minor variations in these categories are possible, in sum they capture all possible morphogenetic behaviours. This article summarises these processes, discusses their measurement, and highlights some salient examples.

## Teaser

**Summary:** This Development at a Glance article describes how a cells’ limited repertoire of behaviours provide the basis for quantifying morphogenetic phenotypes.

## Quantifying cell behaviours to capture directional growth mechanisms

### Morphogenesis through the lens of cell behaviours

Traditionally, analysis of morphogenesis has centred on morphogenetic motifs, such as epithelial invagination or mesenchymal condensation (Bard, 1992; Davies, 2013; Keller, 2006). Although morphogenetic motifs are very useful, they are also diverse (e.g. there are many types of epithelial invagination; Pearl et al., 2017) and there is no way to use them systematically or quantitatively. Focusing instead on cell behaviours provides a key to a systematic analysis of morphogenesis. As Lewis Wolpert often stated, cells can do a rather limited number of different things (Wolpert, 1994). This article summarises what those things are and how they combine to produce growth and morphogenesis. [Fig DEV199794F1]

Capturing morphogenesis systematically in terms of cell behaviours is worthwhile because, first, cell behaviours represent a level of causality and mechanism as legitimate as molecular and genetic mechanisms: collective cell behaviours are emergent from, but cannot be directly extrapolated from, solely genetic or transcriptional information. Second, measurements of an incomplete set of cell behaviours (e.g. proliferation differences alone) cannot reveal the cause of phenotypic change, because unmeasured behaviours could be quantitatively, and therefore mechanistically, more significant when comparing different phenotypes.

**Figure DEV199794F1:**
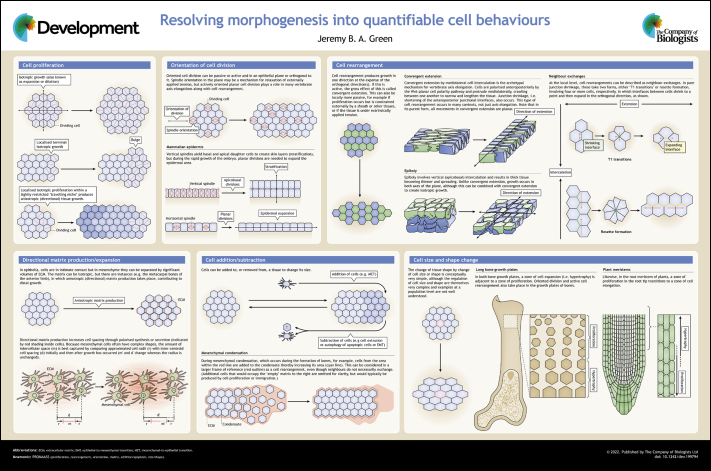


### A cell behaviour framework

A second conceptual lens through which morphogenesis can be analysed is as a vector that can be broken down into its one-dimensional components. All morphogenesis can thus be considered as the sum of ‘growth’ in each dimension. Here, ‘growth’ is used in a very specific sense, meaning an increase in size in a given dimension. This definition allows growth to be expressed as a single number, which can also be a negative number (i.e. shrinkage or narrowing in a given dimension). To avoid the ambiguity between this sense of ‘growth’ and the other sense (i.e. a net increase in tissue mass), the term ‘directional growth’ or the symbol ‘G’ is used henceforth.

Fundamentally, there are six – and only six – cell behaviours that capture how G is generated: (1) (anisotropic) cell proliferation; (2) orientation of cell division; (3) cell rearrangement; (4) directional matrix secretion/expansion; (5) cell addition/subtraction; and (6) cell size/shape change. Minor variations on this list are possible (e.g. separating size from shape or combining orientation with proliferation), but, crucially, this list or its variants are comprehensive and complete. This is important because anatomical phenotypes must, therefore, be attributable to one or more of these processes and, moreover, from an experimental point of view, net G must then be the mathematical product of the length contribution generated in that direction by each of these processes and measuring five of the six plus net G allows the sixth to be inferred. (We have found the mnemonic ‘PROMAASS’, for proliferation, rearrangement, orientation of cell division, matrix, addition/apoptosis and size/shape, is a handy way of referring to this mode of analysis.)

Considering morphogenesis in one dimension and having G as a simple number can be useful in itself. For example, it can be used to discover which of the cellular processes actually contributes most substantively to tissue elongation (Economou et al., 2013). However, if one wants to consider morphogenesis in three dimensions, it is possible to capture G values for each, record their directions and then combine these three vector quantities into what is known in mathematics as a tensor. Tensors can be thought of as a way of writing down a field of vectors (such as a flow field or magnetic field). Similar to vectors, tensors can be decomposed into types of movement (e.g. dilation and shear) that correspond to cell behaviours (e.g. proliferation and rearrangement, respectively). This is the basis of ‘tissue tectonics’ (Blanchard et al., 2009), a pioneering approach in this area, followed by our group's one-dimensional fixed-tissue model (Economou et al., 2013) and more recently by the ‘unified quantitative’ (Guirao et al., 2015) and ‘TissueMiner’ (Etournay et al., 2016) analyses of cell-tracking data.

It is important to point out that a cell-behaviour framework is a kinematic rather than a dynamic one: it quantifies cell behaviours in morphogenesis, but does not capture the forces that generate these behaviours. These forces can be cell-autonomously generated (e.g. active cell migration, programmed cell division orientation), but they can also be non-autonomous (i.e. due to extrinsic forces applied by surrounding tissue or matrix). Thus, it is a substitute neither for biomechanical analysis nor for other analytical approaches, such as continuum models, which ignore individual cells and focus on, for example, the material properties of tissues, or gross scale genetic or physical separation of tissues for determination of autonomy. Nonetheless, capturing the totality of cell behaviours is feasible under a single framework and it defines a massive unrealised research agenda that can make developmental morphogenesis and the comparison of phenotypic mechanisms at the cellular level a tractable problem.

In the sections below, I briefly survey the different cell behaviours to provide examples of their occurrence and experimental quantification for directional growth. This must, by necessity, be a partial selection and I apologise to authors whose work has been omitted.

## Cell proliferation

Although ‘growth’ is often wrongly used to mean just cell proliferation (despite the other processes described below), it is fair to say that most growth – increase in tissue size in one or more directions – has a significant cell proliferation component. Cell proliferation is usually measured indirectly by staining tissue for incorporation of a DNA label, such as BrdU or EdU, and expressing relative proliferation rate as an ‘index’ (i.e. the fraction of nuclei labelled in a given incubation time). Other measures of proliferation include staining for proliferation markers, such as Ki67 (Mki67) or PCNA. All these measures are somewhat indirect and make assumptions about, for example, the constancy of the S phase as a fraction of the cell cycle, which is not always the case (e.g. Brandt et al., 2012; Calder et al., 2013). In principle, double labelling can be used to measure actual cell cycle times (Boehm et al., 2010), but this sometimes gives anomalous results suggesting that the uniformly asynchronous cell cycling assumption may also be wrong. With improved image analysis techniques and genetic lineage labelling, a more direct measurement is feasible, namely counting the number of cells in a given population (e.g. Economou et al., 2013), although sometimes this is not practicable.

We can define proliferation as isotropic (non-directional) cycles of cell division and cell growth, but non-uniformly distributed locally isotropic proliferation can produce directional tissue growth, G. Apical meristems in plants, for example, are zones that extend roots and shoots by proliferation at their tips. The geometry of the shoot or root apex is significant because the proliferating tip cells are only in the central core of a narrowed tip, whereas merely having a terminal growth zone would produce lateral bulging as well as tissue extension. It should be noted, though, that the degree to which the cell divisions within these meristems are actually locally isotropic is unclear (and probably varies between species and organs; Sablowski, 2016). There are many other examples of growth zones (e.g. growth plates in long bones, the mid-palate growth zone), but the fact that many of these grow directionally indicates that proliferation is not the only growth process involved. In the vertebrate limb, for example, although there is a distal-to-proximal growth zone/proliferation gradient, this plays a small or negligible role in the directionality of growth (Boehm et al., 2010). Proliferation is not even necessary for much of morphogenesis: completely inhibiting cell division at early post-gastrulation stages in *Xenopus* embryogenesis still produces morphologically largely normal tadpoles, albeit with fewer cells (Harris and Hartenstein, 1991).

## Orientation of cell division

Although the orientation of cell division is dependent on proliferation to achieve directional growth, its independent regulation justifies its quantification as a separate process. A clear example of directional growth by orientated cell division is that of mammalian epidermis. During early development, planar cell divisions (i.e. those with a mitotic spindle in the plane of the epithelium) generate planar growth as the embryo expands, but this shifts to vertical divisions that generate stratification (i.e. thickening) (Poulson and Lechler, 2010). Orientated cell division is also detectable in mesenchyme (for example, in the developing limb; Boehm et al., 2010). However, it is striking how rarely this mechanism is considered in analysis of mesenchymal growth, and so well-characterised examples are few.

In *Drosophila*, *Xenopus* and mammalian cultured epithelia, planar tissue tension drives changes in cell shape and the disposition of specific structures and proteins at tricellular junctions then act as guides for spindle orientation such that the division relieves the tension (Bosveld et al., 2016; Nestor-Bergmann et al., 2019; Wyatt et al., 2015).

Spindle orientation is not difficult to detect by immunofluorescent staining of the chromosomes and microtubules, although the brevity of anaphase and telophase in mitosis (when the orientation becomes unambiguous) means that a tiny proportion of dividing cells is captured in fixed material.

## Cell rearrangement

Cell rearrangement alone can produce growth in one dimension at the expense of another. The paradigmatic examples of this are in axis elongation by convergent extension (CE) during both *Xenopus* gastrulation and *Drosophila* germband extension (Keller, 2006). In these CE processes, cells intercalate mediolaterally or dorsoventrally, respectively, narrowing the tissue as it lengthens. In *Xenopus*, cell proliferation is suppressed during CE such that tissue narrowing is dramatic, but in other contexts CE takes place together with cell proliferation such that lengthening can take place without concomitant narrowing. CE is widespread in development. Elongation and narrowing of kidney tubules (Lienkamp et al., 2012), the cochlea of the inner ear (Montcouquiol and Kelley, 2020), the cartilage of the jaw (Rochard et al., 2015), the invagination of the molar tooth bud (Panousopoulou et al., 2016), the *Drosophila* hindgut (Iwaki et al., 2001) and the long bone growth zones (Li and Dudley, 2009) are just some examples. CE uses the Wnt planar cell polarity pathway, which is required for the orientation of cell protrusiveness, i.e. mediolaterally in the case of axis elongating CE (Keller, 2006). Intriguingly, planar cell polarity molecules are not localised to the protrusions but to the non-protrusive anterior and posterior faces of the cells (Gray et al., 2011). The intercalation process is not well understood at the cell biological level: at the cellular level it involves what looks like shear at cell interfaces, but, when examined at higher resolution, it involves elements of junction (cell–cell interface) shortening as well as protrusion-led cell crawling (Huebner and Wallingford, 2018).

Another example of growth by cell rearrangement is epiboly, which takes place mostly by cell intercalation in the apicobasal (radial, vertical) axis to produce tissue thinning and spreading (Walck-Shannon and Hardin, 2014). It drives the early development of zebrafish, when the embryo spreads down and around the yolk, and wraps the ectoderm (prospective epidermis and neuroepithelium) around the other germ layers (Bruce and Heisenberg, 2020). Epiboly also occurs in other contexts, for example in ventral closure in mammals (Panousopoulou et al., 2016) and in wound healing (Diegelmann and Evans, 2004).

Detecting and quantifying cell intercalation is non-trivial. Cell intercalation can be observed if individual cells can be live-imaged, for example in whole *Drosophila* and zebrafish or in explants of *Xenopus* and mouse. Quantifying the effects of intercalations on overall directional tissue growth is also mathematically non-trivial. If all cells can be tracked, the abovementioned tensor approaches (Blanchard et al., 2009; Etournay et al., 2016; Guirao et al., 2015) can be used. Tensor analysis requires that the tissue is analysed at a multicellular scale and defining the appropriate length scale (tile or voxel size) large enough to capture coordinated rearrangement without losing individual intercalation events. Another approach is to count ‘T1 transitions’, in which quartets of cells exchange neighbours, and rosette formation, in which larger groups of cells come together in one axis to form rosettes that then spread out in the orthogonal axis (Blankenship et al., 2006). Analysis of these topological changes is more vivid and direct than using the tensor approach, although the relationship to net tissue elongation and the other directional processes is less direct.

A method for extracting the contribution of cell rearrangement to directional growth from clonally labelled fixed material is possible in principle. In theory, clonal labels can be rapidly scrambled (Rulands et al., 2018) and must be very small to be interpretable. This is clearly true in some tissues (e.g. Economou et al., 2013), whereas in others, clones remain remarkably distinct even after long post-labelling development periods (e.g. Chabab et al., 2016; Kaucka et al., 2016). It is also the case that rearrangement immediately following cell division cannot be distinguished by clonal labelling from orientation of cell division, so the latter must be measured independently.

## Directional matrix production/expansion

Epithelial systems such as *Drosophila* and cultured cells are easier to analyse than mesenchyme, but because, in such systems, cells are in tight contact with one another, the volume and therefore growth role of extracellular matrix (ECM) in growth are minimal. However, for mesenchymal tissues, such as limb buds, bones, facial prominences and the heart, growth, and specifically directional growth, the physical bulk of ECM is more significant and can account for directional growth. An example of this is the chick metacarpal, in which a systematic analysis has demonstrated that anisotropic production of ECM is the most significant factor in bone primordium elongation, at least during the stages analysed (Li et al., 2015). In the heart, the valves and septa primordia initially consist primarily of ECM as so-called ‘cardiac cushions’, ECM bumps on the walls of the heart tube. These cushions are gradually filled with increasing numbers of cells that ultimately make the dense tissues of the mature heart, but the initial shaping and growth of heart structures clearly depends heavily on directionally controlled ECM production (MacGrogan et al., 2014). Recently, the controlled swelling of ECM has been shown to be important for morphogenesis of zebrafish semicircular canals (Munjal et al., 2021). In this case, the precise directionality of the matrix swelling has been attributed to polarised E-cadherin (Cadherin 1) actomyosin ‘cytocinches’ rather than direction secretion of matrix itself, but clearly the morphogenesis of ECM can be highly directional and have a (likely under-recognised) contribution to complex morphogenesis.

Cell condensation is the reverse of growth by ECM expansion: spaces between cells decrease and presumably the ECM is either degraded or otherwise remodelled. Depending on one's frame of reference, this could be considered negative growth or, more commonly, as growth of the condensate, in which case it is a version of growth by addition (see below).

ECM, by definition, is what is in the spaces between cells, so, in principle, the ECM contribution to growth can be measured by subtracting cell shape from cell spacing. However, mesenchymal cells can often have extremely complex, typically dendritic or stellate shape (Boehm et al., 2010), and so adequately segmenting (demarcating) the local shape average is even in principle non-trivial. However, whatever method is used to estimate cell size and shape, if it remains constant while cell centroids move apart, the change must be in matrix volume.

## Cell addition/subtraction

Tissue can grow by recruitment of cells from surrounding space. Examples of this include *Drosophila* Malpighian (renal) tubules (MacGrogan et al., 2014), condensation of more posterior cells into the pre-somitic plate as the latter extends posteriorly (Lipton and Jacobson, 1974), and condensation of cells into the expanding cranial bones (calvaria) (Hall and Miyake, 2000). It is possible to treat such cell addition, which could equally be called accretion or recruitment, as a cell rearrangement within a larger frame of reference (tissue domain). Thus, for example, the pre-somitic tissue grows by cell addition, but, within the mesoderm as a whole, this is a combination of cell proliferation and rearrangement. The choice of reference frame can be, in this sense, arbitrary, and depends on which processes the investigator is most focused on. The possibility of cell recruitment from outside a given frame of reference does mean, however, that proliferation and total cell number increase may not be the same.

Conversely, cells can be subtracted or lost from tissue. In solid tissue (mesenchyme), cells are lost through programmed death (apoptosis) and local autophagy. In an epithelium, however, cells can be lost not only through apoptosis (usually following extrusion; Rosenblatt et al., 2001), but also by active delamination (i.e. cells leaving the epithelial layer by epithelial-to-mesenchymal transition. The archetypal epithelial-to mesenchymal transition is that of prospective mesodermal cells delaminating from epiblast in amniote gastrulation. Note that this process can be considered cell subtraction if considering only the epiblast, but constitutes a cell rearrangement if viewing the embryo as a whole.

Cell subtraction must be included in analysis of directional growth processes, even though it is ‘negative growth’. In fact, in most developing embryos, apoptosis is relatively rare because embryos are, in general, growing rapidly. However, there are well-known examples of apoptosis in morphogenesis, such as the tissue between the developing digits of most mammals (Suzanne and Steller, 2013). Where delamination has been tracked in *Drosophila* thorax development, for example, cell subtraction is infrequent and not obviously localised or directionally biased (Guirao et al., 2015). Nonetheless, the amount of apoptosis or delamination is rarely quantified sufficiently well and adequately modelled to know how significant these processes are to overall morphogenesis. It should also be noted that, as with cell recruitment, delamination can be considered either as cell subtraction or as cell rearrangement in a larger frame of reference.

## Cell size and shape change

Cell size change has a conspicuous role in morphogenesis in both plants and animals. In almost all plants, there is a clear progression from small stem cells in apical meristems to much larger, vacuolated cells in the differentiated stems and roots, which clearly drives significant gross growth (D'Ario and Sablowski, 2019). In animal development, the extension of long bones is clearly associated with the increase in the size of cartilage precursors, the hypertrophic cartilage cells (Sun and Beier, 2014). In fact, cell size control may be more significant in other contexts than is currently appreciated given that overall body or organ size is always intimately linked to both cell proliferation and cell growth, and the latter is harder to measure than the former. Cell shape has its clearest role in morphogenesis in the context of epithelia. For example, epiboly (cited above as an example of morphogenesis by rearrangement) can also involve cell shape change: in zebrafish epiboly, in particular, the spreading of cells over the yolk involves cells changing from an approximately cuboidal to squamous shape (Kane et al., 2005).

## Putting the processes together

In summary, the processes of proliferation, rearrangement, orientation of cell division, (polarised) matrix secretion, apoptosis/addition and size/shape must combine to make the net total directional positive or negative directional growth (G) of any tissue in development. These quantities must be multiplied with one another, or added in log space, because each has a ‘fold’ effect on size. For ease of understanding, they have been presented and discussed here largely as one-dimensional quantities (scalars with a direction assumed), but a cell behaviour framework is more general and can be implemented elegantly in two or three dimensions simultaneously using tensors. As mentioned above, tensors are vector-like matrices that mathematically describe fluid flow-like physical transformations, such as dilation and shear, but can be adapted to correspond specifically to each of the cell behaviour quantities itemised in this article (Blanchard et al., 2009; Etournay et al., 2016; Guirao et al., 2015).

Some of the quantities can be combined together in ways that may make measurement easier. For example, cell proliferation plus addition/subtraction is a measure of total cell number change over time, and cell size change plus cell matrix change represents cell packing density change. With cell number and packing density relatively easily measured, the remaining changes are attributable to cell division orientation and cell rearrangement. Of course, cell number and packing measurements alone reveal less about cell behaviours than would the additional segmentation of cell shapes, which distinguish cell size/shape from cell matrix, the measurement of proliferation, etc. The point here is that all of these quantities need to be considered collectively and in an axis of interest to understand morphogenesis in that given axis. Considering multiple axes simultaneously thus requires something equivalent to tensors.

Although the mathematics of tensors may be fearsome to non-mathematical biologists (it is said that even Einstein had trouble with tensors), the power of modern computing and software and the increasing number of computational biologists able to wield these should enable this approach to be widely applied. In fact, the concept of tensors is much simpler than the calculations; it is an array of vectors specified by their location, direction and magnitude. The beauty of using tensors is that, as mentioned above, they can be freely decomposed into the relevant cell behaviours and combined (by matrix multiplication or addition in log space) to calculate the separate and combined morphogenetic effects of these behaviours, respectively. Meanwhile, when growth in just one direction is the key biological question, the one-dimensional approach is both intuitive and accessible.

There is still a great deal of work to be done in improving methods for capturing the processes summarised in this article, particularly for inferring them from fixed tissue where imaging is not practical. There is also room for making the analysis software and data display more user-friendly, accessible, robust, and well-maintained. In due course, advances in both areas will help us understand emergent mechanisms of cell behaviour and link gene data to tangible biological form. ‘Phenotyping’ should start to take this framework into account.
